# Our New York Letter

**Published:** 1887-03

**Authors:** 


### OUR NEW YORK LETTER.

BRAIN-FORCING IN THE EDUCATION OF CHILDREN—VIVISECTION DEMONSTRATES THAT THE HEART SHORTENS IN SYSTOLE—MULTIPLE NEURITIS AND ITS RELATIONS TO CERTAIN PERIPHERAL NEUROSES—A NEW ELECTRICAL APPARATUS, ETC.

*To the Editors of the Atlanta Medical and Surgical Journal:*

Dr. W. A. Hammond read a paper recently before the Nineteenth Century Club entitled "Brain-forcing in the Education of Children." He mentioned the case of a girl brought to him with chorea, who carried such an array of text-books in her school-bag that he only wondered she didn't have meningitis. Her brain capital was being used, and not her brain income. School teachers, he said, were apt to forget that children's brains tire very easily, even on subjects which interest them. In an adult an hour of severe mind-work would cause fatigue, and this occurs sooner, and to a greater degree, in a young child. We have societies whose object it is to guard against children being physically overworked, but he said there were no laws to protect their minds. Dr. Hammond believes that a child's systematic education should be conducted through the perspective organs for the first twelve years of life, and that the one who does not look into a book until this age will read better in a year than a child who learned the alphabet at three. A variety of studies at once he compared to first looking through a microscope and then a telescope, and the effect was to wear out the eyes. He believed grammar should only be taken up during the senior year in college, as no child ever learned to speak good English by studying grammar. The only reason why it did no harm was that nobody really understood its rules. A school-child, he said, learns by memory, and this is not knowledge, but a culture gained at the expense of other faculties. Good memory and poor retentive power generally went together. He believed that those who began study after adult life were the ones who made the most of themselves. They study the real objects, and not only a description of them. Dr. Johnson's definition of a net-work was "a reticulated object with interstices between the intersections," but he did not think a child would get a very clear idea from this. Girls, he said, should have a different education from boys, and mathematics was always a failure in the former.

Miss M. E. Tate, principal of one of our grammar schools, then replied to these remarks. She praised the public school system and admitted its faults, but did not consider them sufficient to condemn the system. She did not believe children were injured in the schools. One case that came under her notice was that of a strong, healthy girl who complained that the work was too arduous and left school. A few days later Miss Tate received her wedding cards. She said she would find school-work herself arduous under such circumstances. Most physicians, she said, knew that the nearest way to a mother's heart was to say to her sick child, "You are not to blame." A doctor had said this of her once, but he saw nothing of the candy and pickles she had been eating. She believed that our school children have not enough to do.

Notwithstanding the vigilance of the officers of the Society for the Prevention of Cruelty to Animals, vivisection continues to be practiced occasionally in our medical colleges. Professor J. G. Curtis, lecturer on physiology at the College of Physicians and Surgeons, lately gave a very interesting demonstration in support of his belief that the heart shortened during its systole. The college janitor first ascertained that none of Mr. Berg's officers were present, and then Dr. Curtis proceeded with his lecture. He reviewed the standing of different physiologists, and quoted numerous experiments made to show that the heart did shorten. A living calf held quiet by straps, in a trough, was brought in and etherized. The doctor then dissected away part of the anterior thoracic wall, exposing the heart, covered by the pericardium, and the lungs to view. During inspiration the lungs nearly covered the heart, but with each expiration it appeared again. The heart was then measured with compasses, and the result obtained was that in systole this organ becomes a little shorter. The animal lived about one hour, and just before death the ascending aorta was tied and the heart removed entire and pinned to a board. It continued to pulsate about one dozen times.

The first lecture of the Middleton Goldsmith course, under the auspices of the Pathological Society, was delivered by Dr. M. A. Starr, the subject being Multiple Neuritis and its Relations to Certain Peripheral Neuroses. In opening the lecture he eulogized Dr. Goldsmith for his active part in the founding of the Pathological Society and for the motives leading to the establishment of this lectureship, which placed the results of individual specialists before the general practitioner in a comprehensive way. A new disease, he said, went through the stages of clinical observation, period of diagnosis, pathological discovery and etiological classification. The first stage of this disease began in 1822, when a Boston physician described a case which we now recognize to be multiple neuritis. After this, isolated cases were reported from time to time, presenting as symptoms some of the following: Pain, paralysis, muscular atrophy and contraction, anæsthesia, formication, hyperæsthesia, sweating, œdema, inco-ordination, fever, loss of tendon reflex and faradic contractility. All these cases were attributed to spinal lesions until one occurred in 1864, where an autopsy revealed wide-spread degeneration of the peripheral nerves, the spinal cord being normal. From this time numerous cases were observed with autopsies which confirmed the diagnosis of multiple neuritis. Nearly all those reported were fatal, but Dr. Starr said that recoveries frequently occurred. After reviewing the normal anatomy of a nerve, he spoke of the changes produced by inflammation. As the best authorities differed on this subject, Dr. Starr concluded that, under different circumstances, different processes occurred. There were two forms of nerve degeneration—a parenchymatous inflammation, only recognized by the microscope, and a diffuse inflammation where the gross appearances indicated congestion. These changes were more intense in the peripheral nerves. In some cases normal segments of a nerve alternated with abnormal portions.

Regeneration might accompany degeneration or follow it, and the time required to complete the process varied. The shortest period was two months, but six months or more were often necessary. Since 1883 about 100 cases have been observed. Diseases formerly supposed to be of spinal origin were now known to arise from peripheral lesions. One was an ataxia from alcohol or arsenic, which resembled tabes. Cases which were thought to have been anterior polio-myelitis are now known as peripheral diseases. Some of the so-called neuroses are also due to neuritis. As instances, he mentioned "numb fingers," "intermittent paralysis," and many indefinite nervous symptoms. The causes of neuritis were said to be mineral poisons, acute diseases, as diphtheria and also malaria. Bæilli produced the epidemic form of the disease known as beri-beri. Cold and over-exertion were also causes of neuritis, and tuberculosis caused a predisposition. The diagnosis, prognosis and treatment were reserved for another lecture.

The electrical apparatus which was used in President Garfield's case has lately been improved upon by Prof. Bell and Dr. J. H. Girdner, of this city. The instrument was exhibited before the Academy of Medicine recently. It is now so arranged that when the explorer is brought near any metallic substance a sound is produced in another part of the apparatus similar to a telephone receiver. Dr. Girdner moved the explorer over the breast of Colonel B. F. Clayton and located a bullet which wounded this soldier at the battle of Cedar Mountain. By another attachment an electrical probe can be made which produces a loud click on touching metal. This was demonstrated on a piece of beef having a bullet imbedded in it. The inventors have no intention of patenting this instrument and the exhibition gave much satisfaction. A. F.

*New York, February*, 1887.

